# Skin innervation and its *in vitro* modeling

**DOI:** 10.3389/fncel.2026.1904623

**Published:** 2026-07-28

**Authors:** Émilie Genin, Lucile Guerber, Elodie Aymard, Jacques Leng, Joëlle Amédée, Alexis Desmoulière, François Berthod

**Affiliations:** 1 LOEX, CHU de Quebec - Laval University Research Center and Department of Surgery, Faculty of Medicine, Laval University, Quebec City, QC, Canada; 2 SILAB, Saint-Viance, France; 3 Laboratory of the Future (UMR 5258), Syensqo, CNRS, University of Bordeaux, Pessac, France; 4 BIOTIS, Inserm U1026, University of Bordeaux, Bordeaux, France; 5 Neuropathies and Therapeutic Innovations (UR 20218), Faculties of Medicine and Pharmacy, University of Limoges, Limoges, France

**Keywords:** CGRP, neuroinflammation, neuropeptides, organ-on-chip, substance P, tissue engineering

## Abstract

Skin innervation consists of myelinated Aβ fibers connected to sensory corpuscles and responsible for the sense of touch, and of free nerve endings including myelinated Aδ fibers and unmyelinated C fibers, that detect temperature and transmit pain signals, in addition to induce neurogenic inflammation through the release of neuropeptides. Moreover, the ability of skin innervation to detect pathogens that trigger or modulate immune reactions, such as TH1, TH2, or TH17 responses, has recently been identified, further growing interest in studying these neuroimmune interactions in the context of skin diseases. To this end, increasing efforts are being devoted to the development of *in vitro* models capable of reproducing the interactions between skin innervation and the various cutaneous cell types in both 2D and 3D culture systems. Combining sensory neurons and Schwann cells in a skin model that must be maintained at the air-liquid interface to promote epidermal differentiation is complex. Furthermore, guiding the migration of neurites through the dermis to make contact with keratinocytes remains a major challenge. The most advanced tissue-engineered 3D models can reproduce innervated skin containing vascular networks and immune cells, while also recapitulating disease phenotypes with cells obtained from patients. Moreover, these models enable real-time monitoring of neuronal electrical activity by connecting neurons with microelectrode arrays. *In vitro* modeling of skin innervation and reproducing the phenotype of skin diseases, should enable major advances in the development of new therapies targeting innervation, with the key advantage to induce far fewer side effects than conventional anti-inflammatory treatments.

## 1 Introduction

The cutaneous nervous system includes sensory and autonomic fibers capable of detecting stimuli ranging from light touch to temperature and pain. Sensory nerves also release neuropeptides such as substance P (SP) and calcitonin gene-related peptide (CGRP), which act not only as pain mediators but also as modulators of inflammation. These molecules influence immune and endothelial cells by promoting vasodilation, vascular permeability, leukocyte recruitment, and immune activation, forming a functional neuroimmune dialogue that shapes inflammatory responses in the skin. Autonomic fibers regulate vascular tone, sweating, and blood flow, thereby contributing to thermal homeostasis and tissue protection (Roosterman et al., 2006).

As the body’s largest sensory and protective organ, the skin serves as a critical interface where the nervous systems play a major role to maintain homeostasis and coordinate functions such as sensory perception, thermoregulation, and immune defense. Cutaneous innervation has been shown to play a major role during wound healing (Lebonvallet et al., 2018). Beyond acting as a physical barrier, keratinocytes actively participate in this system by producing cytokines, chemokines, neurotrophins and neurotransmitters that influence both immune cells and sensory nerve activity (Barbon et al., 2026).

A key component of this integration is neuroinflammation, defined by the dynamic interaction between immune cells and the dense network of C and Aδ sensory nerve fibers innervating the epidermis and dermis. Antigen-presenting cells establish direct contacts with sensory nerve endings, while neuron-derived mediators, including neuropeptides, regulate immune cell maturation, migration, and function (Scholzen et al., 1998). This bidirectional communication enables coordinated responses to infection, injury, and environmental stress (Chiu et al., 2012).

However, the molecular mechanisms underlying neuroimmune interactions in skin diseases are not yet fully understood, largely due to limitations in experimental models. While three-dimensional reconstructed skin and advanced co-culture systems have improved disease modeling, accurately reproducing functional skin innervation *in vitro* remains difficult. This limitation restricts the ability to simulate neuroimmune dynamics and slows down the development of targeted therapies.

To address this gap, tissue engineering approaches that incorporate sensory neurons, keratinocytes, fibroblasts, endothelial cells and immune cells into reconstructed skin models are being explored. Such systems offer valuable tools for studying neuroimmune interactions under controlled conditions (Muller et al., 2018; Vidal Yucha et al., 2019a). Complementary integration with *in vivo* models, including genetically modified animals, is essential for validating mechanisms and accelerating therapeutic translation.

This review summarizes the complex interplay of the nerve network with skin cells and examines current *in vitro* models of cutaneous innervation, evaluating their capacity to replicate these cutaneous interactions. By advancing our understanding of neuroimmune mechanisms and refining experimental models, these approaches may support the development of precision medicine strategies tailored to individual skin biology, ultimately improving the treatment of inflammatory and chronic dermatological conditions.

## 2 Anatomy and physiology of cutaneous innervation

### 2.1 Structure and organization of cutaneous sensory innervation

**Figure 1:**
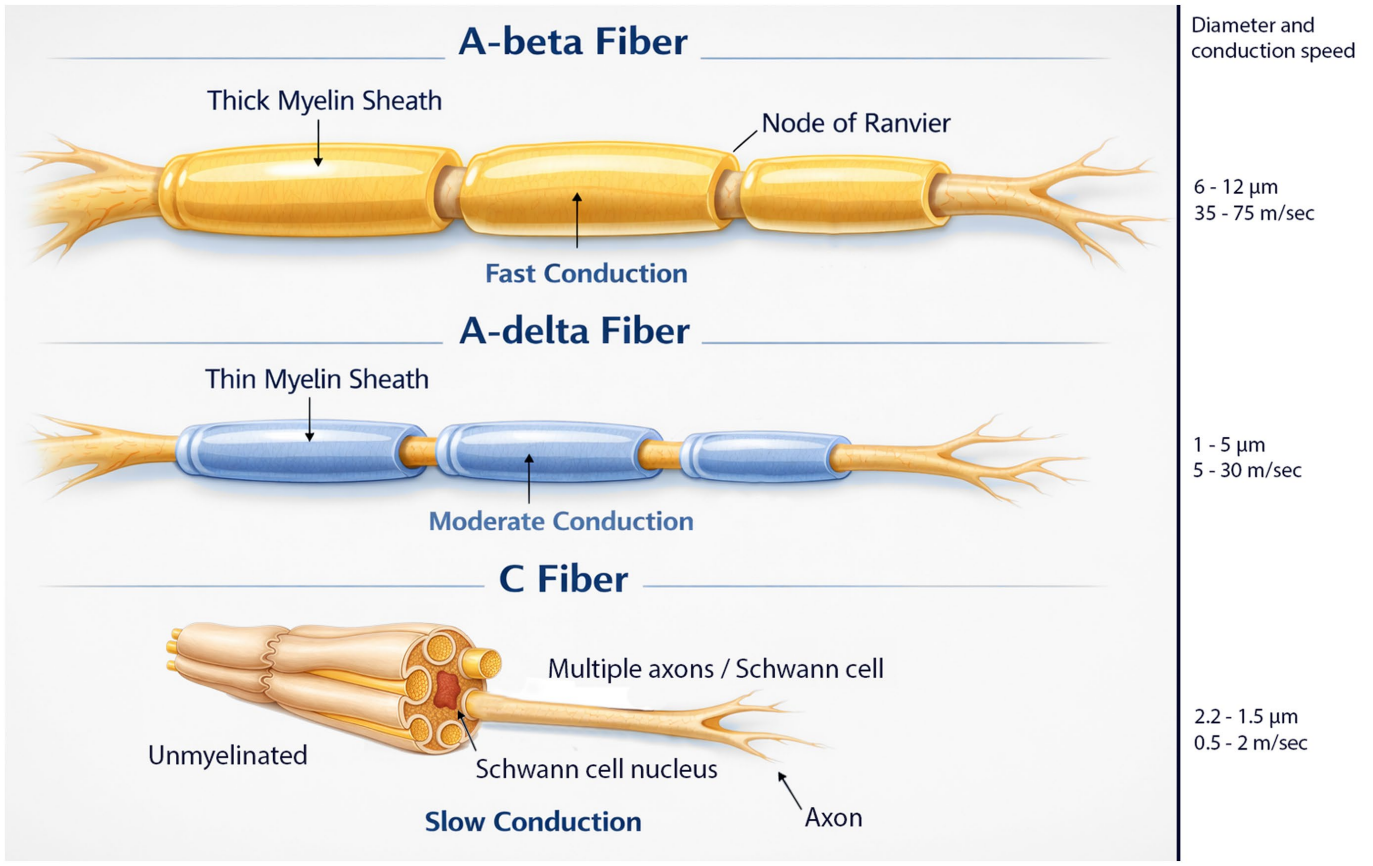
Description of the three different types of nerve fibers in the skin, along with their diameter and the conduction speed of their action potential.

Sensory cutaneous nerve fibers are the peripheral endings of neurons whose cell bodies reside in the dorsal root ganglia (DRG), or spinal ganglia. They transmit signals from sensory receptors in the skin to the central nervous system, where information is relayed through the spinal cord to the appropriate brain integration centers. Autonomic cutaneous nerve fibers are the peripheral endings of neurons whose cell bodies are located in paravertebral ganglia. Cutaneous sensory nerve fibers may be myelinated by Schwann cells, which form myelin sheaths that increase conduction velocity. Larger myelin sheath diameters are associated with faster signal transmission. In contrast, unmyelinated fibers are thinner and conduct impulses more slowly. Cutaneous sensory fibers are broadly classified into three major groups with distinct, although partially overlapping, functions: the Type C, A delta (Aδ) and A beta (Aβ) fibers (Harper and Lawson, 1985) (Figure 1).

Type C fibers are thin unmyelinated sensory nerve endings with slow conduction velocities (0.2–2 m/s) that constitute approximately 80% of the somatosensory cutaneous innervation and subdivide into different subtypes (Ackerley and Watkins, 2018; Niimi et al., 2022). Peptidergic C fibers release neuropeptides including CGRP and SP, non-peptidergic fibers are identified by Isolectin B4 and glial cell line-derived neurotrophic factor family receptor alpha 2 (GFRα2) expression (Jeon et al., 2024); C-tactile fibers (CT) are involved in affective touch and activated by gentle stroking at 1–10 cm/s (Case et al., 2023), and mechano-insensitive C fibers (CMi) are identified by oncostatin M receptor (OSMR) and somatostatin (SST) expression that remain silent under normal conditions but display spontaneous activity in neuropathic pain (Korner et al., 2026).

Aδ fibers are thinly myelinated with diameters of 1.69 μm and conduction velocities of 5–30 m/s (Niimi et al., 2022), mediating fast pain and temperature detection through distinct molecular subtypes: Aδ-COOL expressing Transient Receptor Potential Melastatin 8 (TRPM8) for cold sensation, Aδ-Transient Receptor Potential *Vanilloid 1* (TRPV1) for heat detection, and Aδ-low threshold mechanoreceptors (LTMRs) expressing the *Piezo-type mechanosensitive ion channel component 2* (PIEZO2) for mechanosensation (Korner et al., 2026).

Aβ fibers are large-diameter, heavily myelinated nerve endings with conduction velocities exceeding 30 m/s, traditionally associated with innocuous mechanosensation but now recognized to have more diverse functions (Handler and Ginty, 2021) (Figure 1).

### 2.2 Cutaneous sensory modalities

#### 2.2.1 Thermoregulation and autonomic innervation

Thermoregulation represents a primary function of autonomic skin innervation, with increased cutaneous blood flow and sweating being essential for adaptation to warm temperatures and exercise, and vasoconstriction in cold environment. Autonomic cutaneous fibers are predominantly unmyelinated C-fibers with mean conduction velocities of 0.78 ± 0.12 m/s (Schmelz et al., 1998; Glatte et al., 2019). Sympathetic noradrenergic fibers expressing tyrosine hydroxylase, dopamine beta-hydroxylase and neuropeptide Y (NPY) are abundant around arterioles and arrector pili muscles. Sympathetic cholinergic fibers expressing vesicular acetylcholine transporter, CGRP, SP and vasoactive intestinal peptide (VIP) predominantly innervate sweat glands (Uno, 1977; Ruocco et al., 2002; Donadio et al., 2019). Sympathetic activation causes vasoconstriction through release of noradrenaline, NPY, and ATP (Cracowski and Roustit, 2020). Noradrenergic vasoconstrictor nerves are tonically active in normothermic environments and increase activity during cold exposure (Charkoudian, 2003, 2010). Pilomotor function is mediated by sympathetic adrenergic innervation of arrector pili muscles (Donadio et al., 2019). Active vasodilation in non-glabrous skin is mediated by a sympathetic cholinergic system involving acetylcholine, VIP, and pituitary adenylate cyclase-activating peptide (PACAP) (Cracowski and Roustit, 2020). This active vasodilator system is only activated during increases in body temperature from heat exposure or exercise (Charkoudian, 2010). Parasympathetic fibers expressing CGRP, SP, and VIP innervate arterioles, sweat glands, and arrector pili muscles and are involved in sudomotor and vasomotor functions (inducing sweating and vasodilation) (Donadio et al., 2019; Hijazi et al., 2020). In parallel, stress-induced sympathetic nerve hyperactivation drives hair greying through depletion of melanocyte stem cells caused by the release of noradrenaline (Zhang et al., 2020). Emotional stress also activates neurogenic inflammation through release of SP and CGRP in the skin, and its content of sensory fibers via nerve growth factor (NGF) up-regulation (Joachim et al., 2007).

#### 2.2.2 Sense of touch perception

**Figure 2:**
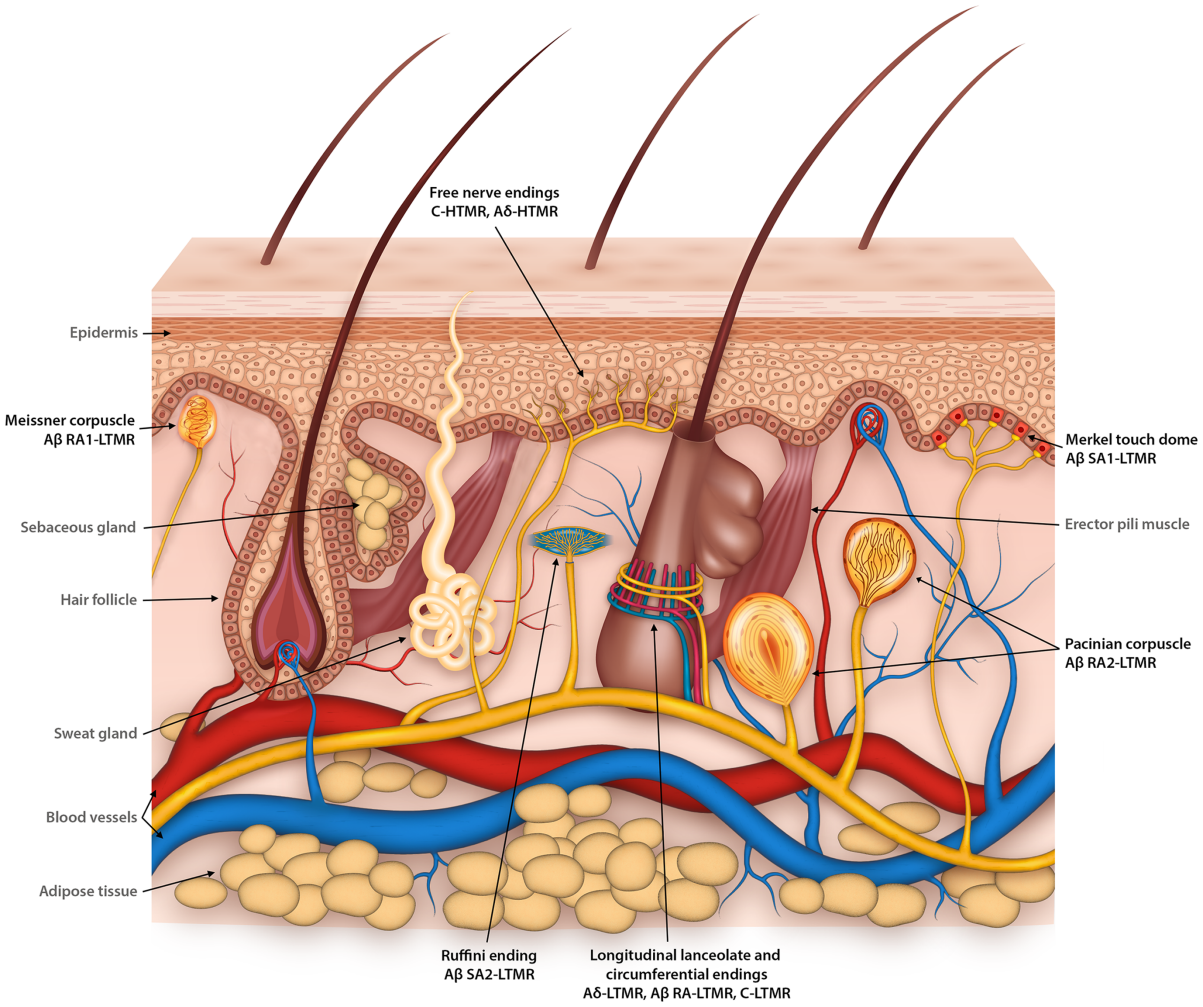
Schematic representation of the nervous network of the skin, composed of Aβ sensory fibers connected to corpuscles or Merkel cells, and of free nerve endings including C and Aδ fibers. The hair follicles are surrounded by the three types of Aβ, Aδ and C fibers. HTMR, high threshold mechanoreceptor; LTMR, low threshold mechanoreceptor; RAI, rapidly adapting type 1; RAII, rapidly adapting type 2; SAI, slowly adapting type 1; SAII, slowly adapting type 2.

Tactile perception has long been thought to be driven by the nerve fibers Aβ for tactile perception, Aδ for cold, puncture senses, and mechanonociception, and C for temperature sensing and pain. It is now recognized that these 3 types of fibers participate together to tactile perceptions, in cooperation with keratinocytes and hair follicles. Aβ fibers are essential for the discriminative aspects of touch through two types of mechanoreceptor units: slow-adapting (SA) and fast-adapting (FA), both being LTMRs. These fibers are connected to corpuscles designed to discriminate specific mechanical stimuli: Meissner corpuscles are arranged like a stack of plates, mainly involved in vibration perception; Pacini corpuscles are organized in onion layers, mainly involved in pression perception; Ruffini corpuscles are arranged along collagen fibers, mainly involved in stretching perception (Rowe et al., 2005; Suazo et al., 2022). SA units remain active throughout the entire duration of mechanical contact, whereas FA units respond primarily to its onset and termination. SA-I and FA-I units have small receptive fields and mediate fine spatial resolution through their association with Merkel cell–neurite complexes (touch domes) and Meissner corpuscles, respectively, both located in or near the basal layer of the epidermis. In contrast, SA-II and FA-II units possess larger receptive fields and are associated with Ruffini endings and Pacinian corpuscles, respectively, which are found in the deep reticular dermis (Figure 2). During object manipulation, FA-I and SA-I units respond prominently to initial skin deformation and changes in grip force, whereas FA-II units respond mainly to mechanical transients and vibration, and SA-II units maintain tonic responses during sustained static conditions (Vallbo and Johansson, 1984; Zimmerman et al., 2014). Recent evidence shows that SA-II and fast-adapting hair follicle afferents reliably differentiate social touch expressions with high accuracy, while FA-II and other subtypes show lower discrimination capacity (Xu et al., 2025). In addition, molecular profiling reveals multiple Aβ subtypes including Aβ-LTMRs for touch and pressure, Aβ-high-threshold mechanoreceptors (HTMRs) expressing Nav1.8 that function as nociceptors, and Aβ-Proprio neurons for proprioception (Yamada et al., 2023; Yamada et al., 2024; Korner et al., 2026) (Table 1).

**Table 1:** Description of the nerve fibers involved in different types of perception, based on Kupari and Ernfors (2023) and Handler and Ginty (2021).

Type of perception	Fiber type	Transducers	End organ location	Type of detection
Temperature	C-Cold	TRPM8	Skin	Cold
	Aδ-TRPM8	TRPM8	Skin	Cold
	Aδ-TRPV1	TRPV1	Skin	Heat
	C-Nociceptor C-Mechano & Heat	TRPV1, TRPA1, TRPM3	Skin	Heat
Pain	C-Nociceptor C-Mechano & Heat	TRPV1, TRPA1, TRPM3, PIEZO2	Skin	Slow pain, chemical, mechanical
	Aδ-H-Nociceptor	TRPV1	Skin	Deep inflammatory pain? Sharp heat pain?
	Aδ-HTMR-Nociceptor	PIEZO2, TRPM8	Skin	Sharp & fast mechanical pain, hair pull pain, noxious cold?
Sense of touch	C-Mechano & Heat	PIEZO2	Skin	Touch
	C-Tactile LTMR		Hairy skin	Pleasant touch
	Aδ-LTMRs		Hairy skin	Hair deflection
	Aβ SAI-LTMR		Merkel cells	Touch (indentation)
	Aβ SAII-LTMR		Ruffini corpuscle	Touch (stretch)
	Aβ RAI-LTMR		Meissner corpuscle	Touch (indentation and vibration)
	Aβ RAII-LTMR		Pacinian corpuscle	Touch (indentation and vibration at high frequency)
	Aβ field-LTMR		Circumferential ending around hair follicles	Skin stroke
	Aβ RA-LTMR		Lanceolate ending around hair follicles	Hair deflection, skin stroke and indentation
	Aβ-HTMR Aδ-HTMR C-HTMR		Glabrous/hairy skin	High force indentation and hair pull
Itch	C-Mechano & Pruriceptor	PIEZO2, MRGPR-D	Hairy/glabrous skin	Mechanical, itch
	C-Pruriceptor	MRGPR-A3, HRH1	Hairy/glabrous skin	Itch
	C-Pruriceptor & Warmth?	HRH1, PIEZO1, TRPM2	Skin	Inflammatory and chronic itch
Proprioception	Aδ-Proprioceptor	PIEZO2	Skin, muscles. Joints	Touch, proprioception
	Aβ-LTMRs Proprioceptor	PIEZO2	Skin, muscles. Joints	Touch, proprioception

HTMR, high threshold mechanoreceptor; LTMR, low threshold mechanoreceptor; RAI, rapidly adapting type 1; RAII, rapidly adapting type 2; SAI, slowly adapting type 1; SAII, slowly adapting type 2; TRPV1: Transient Receptor Potential Vanilloid 1; TRPM8: Transient Receptor Potential Melastatin 8; TRPA1: Transient Receptor Potential Ankyrin 1; MRGPR: Mas-related G-protein coupled receptor; HRH1: Histamine receptor H1.

Type C-tactile fibers are LTMRs maximally activated by gentle, caress-like contact and temperatures similar to human skin (approximately 32 °C). C-tactile fibers show high sensitivity to small skin displacements, particularly during dynamic, moving touch. They terminate around hair follicles, though they remain functionally relevant even in glabrous skin where they are scarce (Ackerley, 2022; Case et al., 2023).

#### 2.2.3 Temperature perception

C-fibers serve as the primary mediators of warm temperature detection in human skin, through the activation of the temperature-sensitive ion channels Transient Receptor Potential Melastatin 2 (TRPM2) for innocuous warmth, and TRPV1, TRPM3 and Transient Receptor Potential Ankyrin 1 (TRPA1) for noxious heat (Kashio and Tominaga, 2022). These unmyelinated fibers conduct at approximately 0.8–1.0 m/s and include functionally distinct subtypes: C warm receptors for innocuous warming, mechano-heat-responsive units (CMH) with thresholds at 40.7 °C, heat-only responsive units (CH) activating at 41–48 °C and Aδ fibers with thermal activation thresholds of 46.9 ± 1.7 °C (Schmelz et al., 1997; Weidner et al., 1999; Plaghki et al., 2010). Cold detection primarily involves thinly myelinated Aδ-fibers with detection thresholds around 25 °C and optimal activation at 20–28 °C, conducting at approximately 12–13 m/s through activation of TRPM8 (Kashio and Tominaga, 2022; Lithfous et al., 2022). However, specialized C-fiber populations also contribute to cold sensation, including Type 2 C-fibers with bimodal thermoreceptive properties responding to both cooling and heating and cold-specific C-fibers activating at 29.4 ± 2.0 °C (Campero et al., 2001; Campero et al., 2009). Regional specialization occurs with glabrous skin showing abundant C warm receptors and fewer Aδ heat nociceptors than hairy skin, while molecular mechanisms involve TRPM8 in C-fibers for innocuous cold and TRPV1/TRPA1 for heat nociception (Courtin and Mouraux, 2022) (Table 1). However, other cutaneous cell types can participate in heat perception, such as keratinocytes through Transient Receptor Potential Cation Channel Subfamily V Member 3 (TRPV3) activation that triggers CGRP release and vasodilation (Peier et al., 2002; Fromy et al., 2018).

#### 2.2.4 Pain perception

Aδ fibers serve as the primary mediator of acute, first pain in human skin, characterized by pricking sensations with noxious heat and rapid conduction velocities of approximately 13 m/s (Plaghki et al., 2010; Churyukanov et al., 2012). These thinly myelinated fibers are the main peripheral mediators for perception of brief noxious stimuli and dominate acute mechanical pain (Magerl et al., 2001; Nahra and Plaghki, 2003). C-fibers, with slower conduction velocities, mediate second, prolonged burning pain (Plaghki et al., 2010; Tzabazis et al., 2011; Hu et al., 2014). However, C-fiber populations exhibit critical functional heterogeneity: mechano-heat-responsive units (CMH, 45% of C-fibers) contribute to acute pain encoding, while mechano-insensitive populations (CMi, 24% of C-fibers) play central roles in chronic pain states through sensitization mechanisms (Schmidt et al., 1995; Henrich et al., 2015). TRPV1-positive C-fibers are the main inducers of both homotopic and heterotopic pain-long-term potentiation (reducing by 71 and 92%, respectively, when blocked), establishing them as primary drivers of chronic pain amplification (Henrich et al., 2015). Peripheral sensitization involves increased nociceptor responsiveness to inflammatory mediators, while central sensitization reflects enhanced synaptic transmission and reduced inhibition in the spinal cord and brain, both essential for acute pain to become chronic (Cheng and Ji, 2008; Latremoliere and Woolf, 2009). This transition from acute to chronic pain involves also fiber-specific temporal patterns: Aδ sensitization manifests within 24 h and remains spatially restricted, while C-fiber alterations include peripheral sensitization of mechano-insensitive units and development of spontaneous activity that extends beyond injury sites (Slimani et al., 2018; Nemenov et al., 2022). A*β* fibers are not primarily nociceptive but mediate allodynia to light touch in secondary hyperalgesia through low-threshold mechanoreceptor pathways (Ziegler et al., 1999). In addition, they play a paradoxical role in mechanical hyperalgesia. They transmit touch-evoked pain locally but can alleviate chronic pain when activated globally (Gautam et al., 2024).

#### 2.2.5 Itch perception

Itch perception is mainly responsible for detection of low-severity danger signals, such as skin contact with allergens, or a mosquito bite. The response to these signals is an urge to scratch to eliminate the source of the itch. The trigger for itching is often a biochemical molecule, such as proteases released during an insect bite or histamine, rather than a physical, chemical, or mechanical trigger as in the case of pain. However, itch is limited to irritation of the skin, whereas pain may affect all parts of the body, and both can be activated by the same TRP channels (Ross, 2011). Histaminergic itch relies predominantly on mechano-insensitive C-fibers via the histamine receptor 1 (H1R) with particularly slow conduction velocities (mean 0.52 m/s) and large innervation territories, also sensitive to prostaglandin E2, acetylcholine and capsaicin (Handwerker, 2010). Non-histaminergic itch recruits both CMi and Aδ fibers depending on the specific pruritogen, such as cowhage spicules, chloroquine, bilirubin or β-alanine, acting through TRPV1, TRPA1, Mas-related G-protein coupled receptor (Mrgpr) and Thymic Stromal Lymphopoietin (TSLP) receptors (Ringkamp et al., 2011; Tsagareli et al., 2023). Indeed, in neurons expressing MrgprA3 and TRPV1, capsaicin exhibited itch, but not pain (Han et al., 2013). Of notes, itch sensation can be relieved by scratch, heat, coolness or menthol through activation of spinal inhibitory B5-I interneurons that release dynorphin (Kardon et al., 2014).

### 2.3 Neurogenic inflammation

Normal tissue response to injury involves an axonal reflex in which influxes triggered in the sensory nerves by the injury return in a retrograde (antidromic) direction to other branches of the sensory nerves, leading to secondary sensitization of neighboring nociceptors and progressive spread of hyperalgesia (Fitzgerald, 1979). However, secondary hyperalgesia appears to involve integrated peripheral and central mechanisms rather than the axonal reflex alone (Woolf, 1983). Neurogenic inflammation in skin is predominantly triggered by activation of C and Aδ nerve fibers through thermal, physical and chemical stimuli, inducing the release of neuropeptides upon stimulation of TRPV1 nociceptors. Neuropeptides activate distinct signaling cascades in target cells: SP triggers Nuclear Factor κB (NF-κB) and Nuclear Factor of activated T-cells (NF-AT) pathways in endothelial cells to upregulate adhesion molecules, while both SP and CGRP activate Extracellular signal-regulated kinases 1/2 (ERK1/2) and c-Jun N-terminal kinase (JNK) Mitogen-Activated Protein Kinase (MAPK) pathways in keratinocytes to drive inflammatory mediator production (Lindsey et al., 2000; Shi et al., 2013; Marek-Jozefowicz et al., 2023). The neuropeptide Pituitary Adenylate Cyclase-Activating Polypeptide (PACAP) released by sensory neurons promotes dendritic cell migration through Pituitary Adenylate Cyclase-Activating Polypeptide Type I (PAC1) receptors, linking neurogenic signals to adaptive immunity (Yamamoto et al., 2021). Peptidergic neurons promote mast cell activation and neurogenic inflammation through SP release (Kakurai et al., 2006). In contrast, MrgprD-expressing nonpeptidergic neurons maintain homeostasis by releasing glutamate triggering kainate receptors to suppress mast cell hyperresponsiveness (Zhang et al., 2021). These nerve fibers release CGRP to stimulate the IL-23/IL-17 pathway, inducing type 17 immunity that manifests as psoriasiform inflammation or antimicrobial defense depending on context (Riol-Blanco et al., 2014; Cohen et al., 2019).

### 2.4 Neural regulation of the immune reactions and its sensing of pathogens

Cutaneous sensory neurons detect multiple classes of pathogens through specialized receptor, such as TRP channels, purinergic P2X channels, mechanosensitive ion channels, G-protein-coupled receptors and cytokine receptors (Udit et al., 2022). Bacterial pathogens include *Staphylococcus aureus* (detected via formylated peptides, alpha-hemolysin toxin, and V8 protease) (Cohen et al., 2019; Deng et al., 2023), *Streptococcus pyogenes* (via streptolysin S) (Pinho-Ribeiro et al., 2018), *Bacillus anthracis* (via Edema Toxin) (Yang et al., 2021), and multiple other species including *E. coli*, *S. pneumoniae*, and *P. aeruginosa* (Chiu et al., 2013). Gram-negative bacteria are broadly recognized through LPS, detected via TRPA1 and LTR4 (Meseguer et al., 2014; Michot et al., 2023). Viral pathogens include *herpes simplex virus type 1* (HSV1) (detected via the Stimulator of Interferon Genes (STING) signaling axis in TRPV1^+^ neurons) and *vaccinia virus* (Filtjens et al., 2021). The yeast *Candida albicans* is detected through extracellular ATP signaling to P2RX2/3 receptors and direct TRPV1^+^ neuron activation (Kashem et al., 2015). The parasitic helminth *Schistosoma mansoni* is detected by TRPV1^+^ and MrgprA3^+^ neurons, though this parasite actively suppresses neuronal activation as an immune evasion strategy (Inclan-Rico et al., 2025). These pathogen-neuron interactions trigger context-dependent immune mechanisms.

#### 2.4.1 Neural modulation of the type 1 TH1 immune response

Sensory and sympathetic cutaneous innervation induce an overall down regulation of the TH1 immune response. *Streptococcus pyogenes* infection is the leading cause of necrotizing fasciitis that can cause rapid death. Its secretion of streptolysin S induce pain by activation of nociceptor neurons, which in turn release CGRP. CGRP inhibits the recruitment of neutrophils and their bacterial killing ability and thus compromise the efficacy of the immune response (Pinho-Ribeiro et al., 2018). Similarly, *Staphylococcus aureus* induces the release of CGRP by Nav1.8^+^ neurons to suppress the recruitment of neutrophils (Chiu et al., 2013) and promoting anti-inflammatory M2 macrophage polarization (Huang et al., 2023). Depleting or inhibiting these neurons can improve control of infection. HSV1 is a neurotropic virus that causes pain but induces a downregulation of neutrophil infiltration in the skin through Nav1.8^+^ neurons to prevent excessive inflammatory reaction. It also promotes a robust CD8 T cell priming and expansion in skin-draining lymph nodes via stimulation of dendritic cells (Filtjens et al., 2021). HSV-1 also activates TRPV1-expressing neurons and limits neutrophils infiltration via SP release, promoting skin healing (Roger et al., 2026). In the context of wound repair, the recruitment of neutrophils, monocytes and macrophages into injured skin is inhibited by Na_V_1.8^+^ nociceptors releasing CGRP. This effect is mediated through thrombospondin-1 secretion to promote an anti-inflammatory and pro-repair phase (Lu et al., 2024). Activation of the sympathetic nervous system via toll-like receptors can also induce a reduced TH1 priming. Cutaneous sympathetic neurons release noradrenaline which activates β2-adrenergic receptors on dendritic cells, leading to a decreased IL12 and increased IL10 secretion (Maestroni, 2006).

#### 2.4.2 Neural modulation of the type 2 TH2 immune response

The allergic TH2 immune response can be induced by TRPV1-positive neural sensing of allergens such as papain, house dust mite cysteine protease, the fungus *Alternaria* or the Diphtheria toxin, which causes SP release (Perner et al., 2020). SP activates dendritic cell migration to the lymph node, TH2 cell differentiation, and mast cell degranulation through the MRGPRX2 receptor (McNeil et al., 2015; Serhan et al., 2019). CGRP induces Langerhans cells to promote TH2 response by inducing up-regulation of IL4 and down-regulation of IFNγ (Ding et al., 2008). CGRP also induces mast cell degranulation (Voisin et al., 2017). Activated mast cells release histamine, tryptase, and serotonin (5-HT), which transmit itch signals through peripheral sensory neurons (Tamari and Ver Heul, 2025). In atopic dermatitis, the release of various molecules such as IL13, IL31 or Thymic stromal lymphopoietin (TSLP) can enhance TRPV1 and TRPA1 expression in sensory neurons, and TRPV3 in keratinocytes, thereby exacerbating pruritus and neurogenic inflammation (Meng et al., 2021). In contrast, the TH2 immune response can be inhibited by MRGPRD-expressing non-peptidergic neurons that release glutamate, suppressing mast cells to maintain skin homeostasis (Zhang et al., 2021; Udit et al., 2022). Finally, the TH2 immune response can be activated by parasympathetic cutaneous innervation via a stimulation of muscarinic and nicotinic receptors by acetylcholine that induces mast cell degranulation and a TH2 polarizing effect on dendritic cells (Bosmans et al., 2017).

#### 2.4.3 Neural modulation of the type 3 TH17 immune response

The most prominent pathway involves CGRP release from activated sensory neurons, which drives IL-23 production from dendritic cells, leading to a TH17 immune response, characterized by the secretion of IL-17A by γδ T cells and subsequent neutrophil recruitment (Kashem et al., 2015; Cohen et al., 2019). This TH17 immune reaction induces an increase in keratinocyte proliferation, leading to an acceleration of corneocyte desquamation that facilitates elimination of dermatophyte fungi (Burstein et al., 2018). However, if CGRP can induce a TH17 activation through cAMP/PKA signaling, it can also inhibit the TH1 immune response by decreasing IL-12 production, both via dendritic cells (Mikami et al., 2012; Mikami et al., 2014). The regulation of this switch between TH17 activation or TH1 inhibition remains unclear. It may be explained by different threshold of neural activation, depending on specific CGRP concentrations, exclusive receptor dynamics or differential binding affinity to CLR/RAMP1 complexes on dendritic cells (Udit et al., 2022). CGRP has also been shown to induce TH17 activation and TH1 inhibition through endothelial cell signaling (Ding et al., 2022). In contrast, CGRP can bind to the CRLR/RAMP3 receptor complex expressed on T cells to enhance the TH1 cell response during viral infection (Hou et al., 2024).

Thus, sensory neurons function as pathogen detectors that calibrate immune responses based on pathogen class and tissue context, balancing pathogen clearance with tissue preservation. This cutaneous neuro-immune modulation can be combined with the role played by peptidergic neurons innervating the lymph nodes (Huang et al., 2021).

### 2.5 Interactions between sensory neurons and skin cells

Cutaneous nerve fibers engage in bidirectional communication with multiple skin cell types. Keratinocytes establish intimate physical contacts with intraepidermal nerve fibers, with nerve endings progressing within keratinocyte cytoplasmic tunnels and connexin 43 plaques localizing at contact sites (Talagas et al., 2020a,b). These interactions are functionally bidirectional: sensory neurons release neuropeptides including SP and CGRP that activate keratinocyte MAPK signaling pathways and induce proliferation (Shi et al., 2013), while keratinocytes actively transduce mechanical stimuli through ATP release that signals via P2X receptors on sensory neurons (Moehring et al., 2018) and can directly initiate action potentials in multiple sensory neuron subtypes (Baumbauer et al., 2015). In addition, keratinocytes can increase nerve fiber growth in the epidermis through the release of neurotrophic factors such as NGF (Roggenkamp et al., 2013) while sensory neurons expressing TRPV1 play an essential role in restoring barrier function following epidermal injury through the potential involvement of somatostatin (Usui et al., 2024).

Sensory neurons increase fibroblast proliferation through release of VIP, CGRP and SP, as well as liberation of matrix metalloproteinases 2 and 9 (MMP2 and MMP9), and their differentiation into myofibroblasts (Cheret et al., 2014; Laverdet et al., 2015). It has been shown that cutaneous innervation is essential for skin tissue repair, underlining close relationships between nerve endings and the different processes and cell types involved in skin repair (Girard et al., 2017; Laverdet et al., 2017).

Melanocytes form synaptic-like contacts with cutaneous nerve fibers (Hara et al., 1996). Innervation is essential for exacerbating pigmentation upon UV-B irradiation (Egriboz et al., 2025). CGRP induces keratinocytes to release melanotrophic factors that stimulate melanocyte proliferation and modulates melanogenesis (Toyoda et al., 1999).

Langerhans cells support MrgprD-expressing nonpeptidergic neurons through neurotrophic factor production, while CGRP from nerve fibers inhibits Langerhans cell antigen presentation (Hosoi et al., 1993; Zhang et al., 2021; Wilcox et al., 2024).

All these bidirectional interactions between sensory neurons, skin cells and the immune system, highlights the need to use *in vitro* models of innervated skin ranging from conventional 2D systems to more complex 3D models to accurately replicate these dynamics and decipher the mechanisms underlying inflammatory skin conditions.

## 3 *In vitro* models of skin innervation

### 3.1 Conventional 2D culture systems

The simplest approach to study the impact of innervation on the skin is to co-culture neurons in a 2D monolayer with keratinocytes, or other types of skin cells. Rather than using cell lines, it is more relevant to obtain freshly isolated human keratinocytes from small skin biopsies, ideally obtained from patients to study a skin disease (Roggenkamp et al., 2012; Pepin et al., 2024). Sensory neurons are usually obtained from mice or rats DRG extraction, at an early stage of embryonic development, such as E12 in mice, before neurites extend out from the ganglion. These neurons are extracted from DRGs along with Schwann cells that are essential to promote axonal migration and long-term survival (Gingras et al., 2003; Cheret et al., 2014). More recently, human sensory neurons, as well as Schwann cells, were differentiated from induced pluripotent stem cells (iPSCs). They were shown to express the neuronal markers neurofilament M, β3-tubulin, and the specific sensory markers BRN3A, and TRPV1. Most importantly, they were shown to release neuropeptides, such as CGRP and SP upon activation with TRP agonists like capsaicin (Muller et al., 2018; Bocciarelli et al., 2023; Louit et al., 2023).

These culture systems are relatively simple to implement over short culture periods and make it possible to highlight specific interactions between neurons and skin cells (Labarrade et al., 2023). They are particularly useful to investigate pathological characteristics of skin cells, such as keratinocytes extracted from patients with atopic dermatitis. These cells overexpress NGF, which induces longer and more branched neurite outgrowth, and increase the proportion of CGRP-positive nerve fibers (Roggenkamp et al., 2012). They also overexpress TSLP that activates TRPA1-expressing neurons to induce itch (Wilson et al., 2013), positioning keratinocytes as drivers of hyperinnervation and itch in atopic dermatitis. Such culture systems were also used to reproduce the ensheathment of intraepidermal nociceptors by keratinocytes, visualizing connexin-43 plaques at keratinocyte-nociceptor contact sites, and demonstrating a functional dialogue in which spontaneous increases in Ca^2+^ in keratinocytes precede neuronal activation, consistent with ATP signaling via connexin-43 junctions, using human iPSC-differentiated sensory neurons and primary keratinocytes (Erbacher et al., 2024).

However, 2D culture systems have major limitations. They do not allow several cell types to be combined in coculture, especially when they do not have the same proliferative or migratory capacities (Moon et al., 2021). Thus, neurons do not proliferate when fibroblasts actively multiply by forming multi-layered sheets, while keratinocytes differentiate when they reach confluency, and endothelial cells need an extracellular matrix to organize themselves into capillaries. Since skin has been the very first organ to be reconstructed *in vitro* in 3D by tissue engineering (Bell et al., 1981), the development of more complex and pluricellular innervated skin substitutes have been a promising field or research in the last two decades (Gingras et al., 2003; Blais et al., 2009; Gagnon et al., 2011; Ahn et al., 2023).

### 3.2 Microfluidics and organ on chips

The microfluidic toolbox is mainly based on compartmentalization on a scale of 0.1–100 μm, meticulous flow management, and the possibility to exert localized stresses. The combination of these three levers has led to the emergence of the organ on chip technology, in which minimal representative models of parts of organs are cultured in 2D or 3D geometries and fed dynamically (Bhatia and Ingber, 2014; Rodriguez-Garcia et al., 2020).

Regarding skin-on-chip, various methodologies were developed, such as diffusion, toxicology or drug efficacy tests, wound healing, repair, inflammation, ageing and shear stress studies, mass transfer applied to skin, heat transfer, and also the specific features of the materials used to manufacture organ on chips (Mohammadi et al., 2016; Sutterby et al., 2020; Ponmozhi et al., 2021; Risueno et al., 2021).

Microfluidics relies on microfabrication of custom fluidic circuits, 2D and 3D chambers and channels and control of the inner flow (Martinez-Rivas et al., 2017). A canonical example of compartmentalization was developed to analyze interactions between neurons and other cell types, based on two small culture chambers (1 μL) separated by a cell-filter (thin and long microgrooves) (Taylor et al., 2003). Neurons are seeded and cultured in one chamber and only neuritic extensions can pass through the microgrooves. The second chamber serves to deliver molecules that can modulate axonal migration or to investigate the interaction between neurites and other cells (Leroux et al., 2020; Leroux et al., 2024; Bessy et al., 2026). These microfluidic culture systems are useful to investigate drug toxicity on neurons and neuronal disease mechanisms. Chemotherapy-induced peripheral neuropathy (CIPN) was recapitulated in vitro through monitoring of electrophysiological changes or neurite outgrowth inhibition detected in a rat DRG nerve on-a-chip microfluidic system exposed to drugs such as oxaliplatin to investigate their toxicity in vitro (Kramer et al., 2020; Pollard et al., 2021; Adhikari et al., 2024; Du et al., 2026).

Neuropathic pain and diabetic neuropathy can also be studied in vitro using primary sensory neurons, iPSC-derived neurons and 3D organoids (Hattangady and Rajadhyaksha, 2009; Zebochin et al., 2024).

Small fiber neuropathy (SFN) in Fabry disease was recapitulated using patient-derived iPSCs differentiated into sensory neurons, compared to isogenic and healthy controls. An accumulation of intra-lysosomal globotriasylceramide was detected in soma and neurites of SFN neurons, as well as a thinning of neurite calibres (Klein et al., 2024).

The use of 3D innervated skin models for these studies would be a major upgrade (Shin et al., 2022). To develop 3D model of skin innervation, pillars were used to physically hold a gel in place. The gel that serves for bulk cell culture can then be put into contact with a medium or other cells. This slab geometry has been used to test wound healing (Biglari et al., 2019), epidermal innervation (Giorgi et al., 2023), or vasculogenesis (Wang et al., 2016).

### 3.3 Tissue-engineered 3D innervated skin models

#### 3.3.1 Conventional 3D models

Many tissue-engineered skin substitutes have been developed over the last four decades, with reconstruction of the dermis remaining the greatest challenge. Early approaches relied on collagen gels populated with fibroblasts, whose contractile activity promoted matrix compaction, onto which keratinocytes were subsequently seeded (Bell et al., 1981). However, a major limitation of this strategy was the lack of active remodeling of the rat- or bovine-derived collagen matrix by human fibroblasts. In contrast, fibroblasts cultured within porous biomaterials such as collagen sponges deposited large amounts of human collagens and extracellular matrix proteins (Berthod et al., 1993; Berthod et al., 1996). This fibroblast-derived matrix was shown to closely resemble the native dermis and was associated with the re-expression of several collagens that are typically lost in conventional 2D cultures (Berthod et al., 1997). Similar benefits were achieved without the use of any scaffold or gel by culturing fibroblasts as monolayers for extended periods in the presence of ascorbate, allowing extracellular matrix accumulation and the formation of fibroblast sheets (Michel et al., 1999; Germain et al., 2002). An alternative approach consists of using native skin explants ex vivo. Although this strategy preserves all resident skin cell populations, including immune cells, its major drawbacks are limited survival in culture and complete denervation of the tissue (Chéret et al., 2022).

The absence of skin innervation can be partially compensated for by supplementing the culture medium with neuropeptides such as SP or CGRP, thereby mimicking neural activation (Muller et al., 2018). But neuropeptides must diffuse from the culture medium into the tissue despite their very short half-life. In contrast, adding neurons in the model to build a network of sensory neurites that extend close to the epidermis offers a major advantage. It enables the release of a more physiological repertoire and concentration of neuropeptides directly in the vicinity of basal keratinocytes following neuronal activation with TRPV1 agonists like capsaicin (Muller et al., 2018; Pepin et al., 2024).

Therefore, the principal challenge in developing innervated tissue-engineered skin lies in the incorporation of a functional nerve network. When skin explants or skin substitutes are simply deposited onto neurons cultured in 2D, axonal migration into the tissue can be observed as a result of neurotrophins secretion by the epidermis. Such nerve fibers have been shown to restore epidermal proliferation, vascular density, and mast cell survival to levels approaching those observed in fresh skin (Chéret et al., 2022). However, this reinnervation process remains limited in both abundance and homogeneity and can only be maintained for relatively short periods (Lebonvallet et al., 2021; Bellantoni et al., 2026). To promote more extensive nerve ingrowth, a two-step strategy has been developed. Sensory neurons isolated from mouse DRGs or differentiated from human iPSCs are first seeded onto a tissue-engineered dermis cultured above a medium supplemented with NGF, thereby creating a gradient that guides axonal migration through the tissue. Subsequently, the innervated dermal equivalent is inverted, and keratinocytes are seeded onto the opposite surface, away from the neuronal cell bodies (Blais et al., 2014). This approach allows numerous neurites to establish close contact with keratinocytes and locally release neuropeptides following TRPV1 stimulation. Moreover, these innervated skin models can be maintained in culture for extended periods exceeding 1 month (Muller et al., 2018; Pepin et al., 2024).

Schwann cells play an essential role in neurite extension within three-dimensional skin equivalents. Their incorporation into reconstructed skin enhances axonal migration both *in vitro* and after transplantation *in vivo* (Blais et al., 2009). While Schwann cells efficiently support axonal growth of mouse DRG neurons in 3D innervated skin models, their absence in sensory neurons derived from iPSCs may compromise the establishment of a nerve network capable of reaching the epidermis. Consequently, the co-generation of Schwann cells alongside iPSC-derived sensory neurons is likely required for efficient tissue innervation (Muller et al., 2018). These iPSC-differentiated Schwann cells were shown to successfully form myelin sheaths around iPSC-differentiated neurons in vitro (Louit et al., 2023).

The complexity of these models has been further increased through the incorporation of specific anatomical niches. Skin appendages, particularly hair follicle structures, act as pro-innervating niches that guide nerve fiber migration both in vitro and following transplantation in vivo (Gagnon et al., 2011).

Sensory neurons also contribute to skin homeostasis and epidermal repair. In an in vitro wound-healing model, sensory neurons accelerated wound closure by approximately two-fold. This effect was mediated by SP, which activates neurokinin-1 (NK1) receptors on keratinocytes, thereby enhancing their migratory capacity without affecting proliferation. Importantly, this response could be abolished by NK1 receptor antagonists (Blais et al., 2014). Furthermore, reinnervated skin explants have demonstrated significant potential for investigating itch and neurogenic inflammation in vitro, revealing distinct response profiles following activation of TRPV1, TRPA1, and PAR-2 (Lebonvallet et al., 2021).

These advanced 3D models also provide valuable platforms for studying complex skin diseases (Schutte et al., 2021). For example, diabetic skin cells exhibit a reduced capacity to promote nerve fiber migration in vitro due to impaired glyoxalase activity, as demonstrated in a 3D skin model (Reichert et al., 2017). To model atopic dermatitis, iPSC-derived itch-sensitive sensory neuron-like cells expressing IL-4 and IL-13 receptors have been generated and shown to respond directly to these cytokines. Their incorporation into collagen-based skin equivalents provides a relevant platform for pharmacological screening in the context of atopic dermatitis (Guo et al., 2022). Psoriasis has likewise been recapitulated using tissue-engineered skin reconstructed from patient-derived cells. These psoriatic models display a hyperproliferative keratinocyte phenotype compared with healthy controls (Rioux et al., 2021). When innervated with mouse DRG neurons, capsaicin-induced neuropeptide release resulted in increased keratinocyte proliferation exclusively in the psoriatic model. This exacerbation of the psoriatic phenotype was attributed to CGRP, as the effect was abolished in the presence of a CGRP receptor antagonist (Pepin et al., 2024) (Table 2).

**Table 2:** Description of the different 3D models of innervated skin.

Type of skin cells	Type of neurons	Additional cell types	Specific parameter	Type of dermal architecture	Objectives	References
Human healthy fibroblasts	Mouse DRG neurons	HUVEC		Collagen sponge	Model to study peripheral nerve growth in vitro	Gingras et al. (2003)
Human healthy fibroblasts and keratinocytes	Mouse DRG neurons	HUVEC, mouse Schwann cells		Collagen sponge	Impact of Schwann cells on neutite growth in vitro and in vivo	Blais et al. (2009)
Mouse healthy fibroblasts and keratinocytes	Mouse DRG neurons	Mouse immature hair follicles		Self-assembled fibroblast sheet	Impact of immature hair follicles on neurite migration	Gagnon et al. (2011)
Human healthy or atopic fibroblasts and keratinocytes	Porcine DRG neurons		Patient-derived cells	Collagen gel	Impact of nerves on keratinocyte growth	Roggenkamp et al. (2013)
Human healthy fibroblasts and keratinocytes	Mouse DRG neurons	HUVEC or HDMEC		Collagen sponge	Impact of innervation in wound healing	Blais et al. (2014)
Human healthy fibroblasts and keratinocytes	Mouse DRG neurons	HUVEC		Collagen sponge	Impact of glycation on the nerve and vascular networks	Cadau et al. (2015)
Human healthy or diabetic fibroblasts and keratinocytes	Porcine DRG neurons		Patient-derived cells	Collagen gel	Impact of diabetes on neurite migration	Reichert et al. (2017)
HaCat cells and healthy human fibroblasts	Rat DRG neurons			Gelatin microbeads	Model of neuronal calcium current generation	Martorina et al. (2017)
Human healthy fibroblasts and keratinocytes	Human neurons differentiated from iPSCs	Human Schwann cells differentiated from iPSCs, HDMEC		Collagen sponge	Model of fully human innervated tissue-engineered skin	Muller et al. (2018)
Human healthy fibroblasts and keratinocytes	Human induced neural stem cells	Human adipose tissue	Hypodermis-derived immune cells	Silk—collagen gel	Model of human innervated tissue-engineered skin with hypodermis	Vidal et al. (2019) Vidal Yucha et al. (2019b)
Human skin explant	Rat DRG neurons			Skin explant	Model to study itch and neurogenic inflammation	Lebonvallet et al. (2021)
Human skin explant	Rat DRG neurons		Human skin-derived mast cells	Skin explant	Model to study interactions between nerves and mast cells	Chéret et al. (2021)
Human healthy fibroblasts and keratinocytes	Human neurons differentiated from iPSCs			Collagen gel	Model of human innervated tissue-engineered skin	Guo et al. (2022)
Human skin explant	Human neural stem cells			Skin explant	Model of reinnervated human explant	Chéret et al. (2022)
Human healthy fibroblasts and keratinocytes	Human iPSC-derived neurosphere			Collagen-fibrin hydrogel	Model of neurite regression induced by capsaicin	Malheiro et al. (2023)
Human healthy keratinocytes	Rat DRG neurons			Microfluidic chip	Model of an innervated epidermal layer in a microfluidic chip	Ahn et al. (2023)
Healthy or psoriatic human fibroblasts and keratinocytes	Mouse DRG neurons		Patient-derived cells	Collagen sponge	Impact of innervation on the psoriatic phenotype	Pepin et al. (2024)
Healthy human fibroblasts and keratinocytes	Rat DRG neurons	Human Schwann cells	Electrical activity recording	Porous gelatin microcarriers	To monitor neuron electrical response with a microelectrode array	Bellantoni et al. (2026)

HUVEC, human umbilical cord endothelial cells; HDMEC, human dermal microvascular endothelial cells.

#### 3.3.2 Current limitation in validation of neuroimmune functions in innervated skin models

To effectively reproduce the neuroimmune interactions whose importance in the study of inflammatory skin diseases is becoming increasingly evident, it is necessary to develop immunocompetent innervated skin models. However, to our knowledge, such models do not yet exist, except for the reinnervated skin explant model, in which resident immune cells, such as mast cells, are preserved (Chéret et al., 2021), and the innervated skin model enriched with hypodermis-derived cells, in which CD68-positive cells have been detected (Vidal Yucha et al., 2019b).

The incorporation of immune cells such as dendritic cells, macrophages, mast cells, and lymphocytes into innervated skin models represents a major challenge, given that these models are already time-consuming to develop and that immune cells generally exhibit limited survival in vitro. More importantly, demonstrating the preservation of their functionality, namely, their ability to respond to stimuli by releasing cytokines and other mediators, remains particularly difficult.

Nevertheless, highly innovative approaches have recently been developed in microfluidic systems to integrate immune cells into tissue models. These models will be discussed in Section 3.3.4.

#### 3.3.3 Bioprinting approaches

3D bioprinting offers promising prospects for reproducing the complex hierarchical architecture of the skin (Pourchet et al., 2017). Recent reviews highlight the predominance of natural polymers in bioprinting full-thickness tissues. While gelatin and alginate provide essential cytocompatibility, their mechanical limitations are frequently mitigated through the formation of composite hydrogels or the incorporation of synthetic polymers (Aljohani et al., 2018). Stabilization of these constructs typically relies on diverse crosslinking mechanisms, including ionic gelation (Ca^2+^), UV radiation, and pH-driven polymerization (GhavamiNejad et al., 2020). These approaches have enabled the successful integration of vascular networks within the dermis (Zhang et al., 2026). Other strategies, such as the use of sacrificial alginate channels, have been proposed to engineer vascular-like networks (Wang et al., 2014). However, bioprinting has not yet been used to successfully develop functional innervated skin models, still favoring microfluidic platforms or conventional scaffold-based approaches.

#### 3.3.4 3D skin-on-chip models

Topologies and 3D geometries are most often set up with gels that are chosen to mimic the extracellular matrix and can be formulated to modulate cell fate. Pre-gel cell suspensions are injected into a chip on one side of a membrane or retained by pillars, left in place to settle and brought into contact with a medium or another cell culture. To add complexity, the gel can be modelled in several ways: molding, 3D printing, subtractive design, etc., and the architectures are designed to be perfusable, deformable under osmotic stress and mechanical forces (Owen and Shoichet, 2010; Zhang and Larsen, 2017). Mesh-like structures with open porosity allow the circulation of a medium or the seeding of other cellular material; conversely, sacrificial structures can be incorporated before the gel has fully set and the lumen is then created when the sacrificial structure is removed (Golden and Tien, 2007). Other important structuring elements such as spheroids and organoids have been successfully integrated and cultured on chip, which is particularly relevant for skin (Fang et al., 2023).

Due to the complexity of integrating neurons with 3D skin constructs, few studies have reported the development of innervated skin-on-chip models. The main challenges include positioning neurons beneath the construct to mimic physiological conditions, while promoting neurite extension toward the epidermis. At the same time, the model must be maintained at an air–liquid interface to promote epidermal differentiation, which limits the supply of culture medium to the basal side only. Consequently, achieving both robust axonal outgrowth and proper epidermal differentiation remains difficult (Ahn et al., 2023) (Table 2).

**Figure 3:**
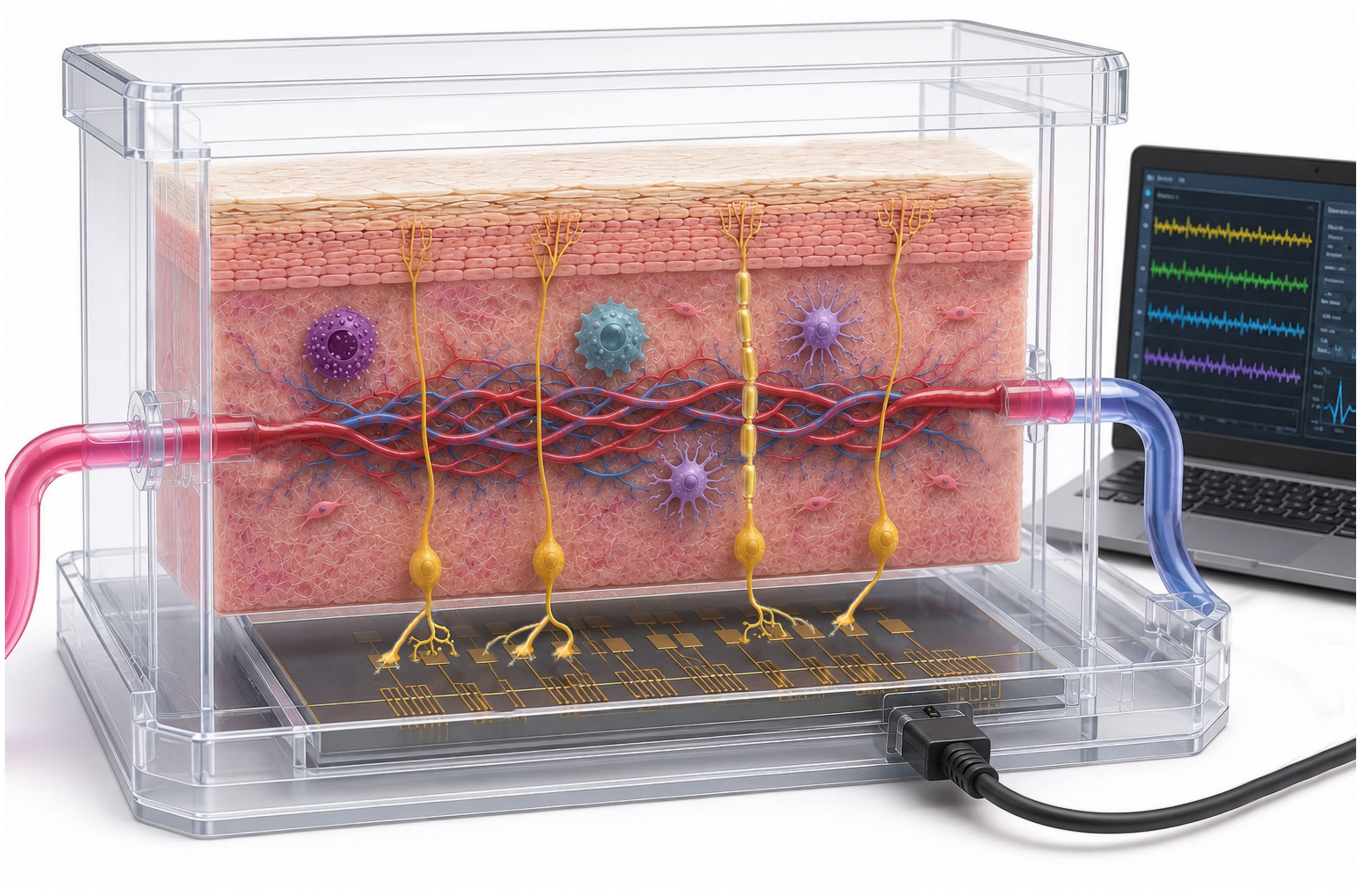
Schematic representation of an ideal tissue-engineered skin model combining an epidermis formed by keratinocytes and a dermis containing fibroblasts, Schwann cells, and immune cells, including dendritic cells, mast cells, and macrophages. The tissue is perfused with culture medium via a microfluidic system connected to a capillary network composed of endothelial cells and pericytes within the dermis. A sensory nerve network, generated from neurons cultured beneath the tissue, extends neurites toward both the epidermis and the basal side of the construct. At the base of the model, these neurites establish connections with a microelectrode array on which the tissue is deposited, enabling real-time monitoring and analysis of neuronal electrical activity.

While innervated skin models can be developed very efficiently by classical tissue engineering approaches, independently of bioprinting and microfluidic chamber culture, a major methodological advance has been achieved through their integration with high-density microelectrode arrays (HD-MEAs). In this configuration, sensory neurons are either cultured directly on the recording electrodes, where they extend neurites into the overlying skin equivalent, or the pre-innervated skin construct is subsequently placed onto the recording surface. In both cases, this setup enables electrophysiological recording of neuronal action potentials in response to topical application of compound on the epidermal surface, thereby providing a functional platform for assessing its nociceptive effects on the skin (Bellantoni et al., 2026). The main limitation of this approach is to restrict the culture medium supply of the tissue to its lateral sides. This restraint could be overcome by a perfusion of medium through a microfluidic system connected to a capillary-like network incorporated within the dermis, which is however difficult to succeed (Wang et al., 2016) (Figure 3). This strategy should make it possible to study pathologies such as diabetic neuropathies, among others, using patient cells. Microelectrode arrays will also make it possible to electrically stimulate neurons, to evaluate their effect in diseases such as psoriasis, rosacea or atopic dermatitis, in which innervation can play a central role.

Finally, while no model of innervated skin has demonstrated an effective neuroimmune interaction, several other types of culture systems have integrated immune cells. In particular, microfluidic systems composed of human capillaries were perfused with various immune cells, such as monocytes-like cells (THP1), dendritic cells or Natural Killer cells (Parlato et al., 2017; van Dijk et al., 2020; Song et al., 2021). This type of model makes it possible, among other things, to study the process of extravasation of immune cells as well as metastatic cells through the endothelial wall (Mondadori et al., 2020). Some 3D skin models were also developed to combine immune cells and endothelial cells to study their impact on wound healing or skin inflammation (Kwak et al., 2020; Attiogbe et al., 2026; Yuan et al., 2026). The integration of immune cells into a model of innervated skin is therefore probably only a matter of time.

## 4 Discussion

### 4.1 Perspectives in the development of 3D innervated skin models

Despite these advances, several technological and biological barriers remain, limiting the full clinical transposability of these models. Neurons derived from iPSCs often display low functional maturity and high inter-individual variability (Schwartzentruber et al., 2018; Kalia et al., 2024). Murine DRG-derived neurons may benefit from higher diversity, and are combined with Schwann cells, but are of animal origin and obtained usually at an embryonic stage of development. The combination of autonomic neurons to the sensory nerve network could be a valuable improvement, especially considering their modulation of the vascular and immune components, but these neurons remain challenging to extract. Most models still lack resident immune cells (Langerhans and dendritic cells, T cells, mast cells), limiting the study of complete neuro-immune interactions (Vidal Yucha et al., 2019a). Some hybrid models (e.g., rat neurons on human skin or PC-12 cells) rise concerns of inter-species compatibility. Problems with imperfect epidermal stratification, scaffold shrinkage and compound absorption by microfluidic chip materials require continuous technical optimization (Bettinger et al., 2005). Despite remaining challenges regarding neuronal maturity, incomplete cellular composition, species heterogeneity, and material constraints, the transition to physiologically relevant innervated skin models offers unprecedented opportunities. While *in vivo* validation remains necessary, these advanced systems are paving the way for a better understanding of chronic skin diseases and the development of targeted therapies. Such therapies could present major advantages, especially in the treatment of inflammatory skin diseases such as psoriasis, atopic dermatitis or rosacea. Indeed, the most severe case of psoriasis can be treated with biological therapies based on anti-TNFα monoclonal antibodies. This approach can be very efficient but induces an immunosuppression that can increase risks of infection and cancer (Solovan and Chiticariu, 2013; Peleva et al., 2018). In contrast, a biological therapy using anti-CGRP monoclonal antibodies to treat migraine has been shown to induce only minor side effects, such as dizziness (Hou et al., 2017). Treatments with antagonists of the neurokinin 1 receptor for SP against atopic dermatitis (Martinez and Philipp, 2016), and anti-CGRP antibodies against psoriasis could be promising therapies, with a topical approach for moderate cases, and systemic treatment for more severe ones.

## 5 Conclusion

Skin innervation is a parameter that has long been neglected in research on skin pathophysiology. This can probably be explained by the challenge it poses to study, but also by the fact that innervation was purely confined to its role in the perception of temperature, pain and the sense of touch. It has become clear in the last 20 years that the modulation of neurogenic inflammation by sensory innervation can play a major role in many skin pathologies (Chiu et al., 2012). In addition, by realizing that sensory nerves can specifically detect many pathogens, and trigger or modulate a specific immune reaction in return, whether it is a TH1, TH2 or TH17 response, skin innervation is now attracting growing interest (Udit et al., 2022). This late interest is reflected in the efforts that have been developed in the *in vitro* modeling of skin innervation. While the vascularization of the skin has generated many studies using bioprinting, hardly any attempt has been made to model innervation by this approach. The use of microfluidic culture techniques has been more common, as it lends itself well to tracking axonal migration along microchannels in 2D culture, but is much more difficult to adapt to a 3D model. Finally, the most advanced models of innervated skin were produced using classical approaches based on biomaterials or skin explants, combining up to five cell types, including neurons, Schwann cells, fibroblasts, keratinocytes and endothelial cells (Cadau et al., 2015; Muller et al., 2018). The next step in the maturation of these models will be to add immune cells, such as dendritic and Langerhans cells, macrophages, *T* cells and mast cells, to be able to produce representative models of different skin diseases, using cells obtained from patient skin biopsies and blood samples (Moon et al., 2021). The final touch in the evolution of these models will be to place them on microelectrode arrays that will allow real-time monitoring of the electrical activity of neurons, depending on the stimuli that can be tested (Bellantoni et al., 2026). These electrodes will also make it possible to induce a depolarization of neurons at will to cause the release of neuropeptides in direct contact with the epidermis, avoiding the toxic effect of an agonist such as capsaicin. Thanks to this type of model, it will then be possible to understand precisely the role played by sensory innervation in several diseases, such as psoriasis, atopic dermatitis, rosacea or neuropathies (Msheik et al., 2022). It will also be possible to use these models as screening platforms for new therapeutic molecules.
